# Extracellular vesicles from the trematodes *Fasciola hepatica* and *Dicrocoelium dendriticum* trigger different responses in human THP‐1 macrophages

**DOI:** 10.1002/jev2.12317

**Published:** 2023-04-19

**Authors:** Christian M. Sánchez‐López, Aránzazu González‐Arce, Carla Soler, Víctor Ramírez‐Toledo, María Trelis, Dolores Bernal, Antonio Marcilla

**Affiliations:** ^1^ Área de Parasitología, Departament de Farmacia i Tecnologia Farmacèutica i Parasitologia Universitat de València Burjassot (Valencia) Spain; ^2^ Joint Research Unit on Endocrinology, Nutrition and Clinical Dietetics Health Research IIS La Fe‐Universitat de València Valencia Spain; ^3^ Food & Health Lab. Instituto de Ciencia de los Materiales Parque Científico, Universitat de València Valencia Spain; ^4^ Veterinari de Salut Pública Centre de Salut Pública de Manises Valencia Spain; ^5^ Departament de Bioquímica i Biologia Molecular, Facultat de Ciències Biològiques Universitat de València Burjassot (Valencia) Spain

**Keywords:** *Dicrocoelium dendriticum*, *Fasciola hepatica*, helminth EVs, macrophage activation, monocyte migration, SEC isolation

## Abstract

Extracellular vesicles (EVs) released by the helminths *Dicrocoelium dendriticum* and *Fasciola hepatica* are important modulators of the host immune response, contributing to the establishment of the infection. Monocytes and, in particular, macrophages are major regulators of the inflammatory response and are likely responsible for the phagocytosis of most of the parasite EVs. In this study, we isolated EVs from *F. hepatica* (*Fh*EVs) and *D. dendriticum* (*Dd*EVs) by size exclusion chromatography (SEC) and characterized them by nanoparticle tracking analysis, transmission electron microscopy and LC‐MS/MS, and analyzed the cohort of proteins. The treatment of monocytes/macrophages with *Fh*EVs, *Dd*EVs or EV‐depleted fractions from SEC, demonstrated species‐specific effects of the EVs. In particular, *Fh*EVs reduce the migratory capacity of monocytes and the analysis of the cytokine profile showed that they induce a mixed M1/M2 response, exerting anti‐inflammatory properties in Lipopolysaccharide‐activated macrophages. In contrast, *Dd*EVs do not affect monocyte migration and seem to have pro‐inflammatory properties. These results correlate with the differences in the life cycle of both parasites, suggesting different host immune responses. Only *F. hepatica* migrates to the bile duct through the liver parenchyma, driving the host immune response to heal deep erosions. Furthermore, the proteomic analysis of the macrophages upon *Fh*EV treatment identified several proteins that might be involved in *Fh*EV‐macrophage interactions.

## 1 INTRODUCTION

Parasitic helminths affect nearly one third of the human population and they are extraordinarily prevalent in low‐income countries (Jourdan et al., 2018). An estimated 1066 million people are at risk of infections by food‐borne trematodiases, representing a serious public‐health problem (Fürst et al., 2012). In addition, the parasitic helminths are a serious problem for livestock worldwide, causing important economic losses by affecting their feed intake, fertility and milk production (Charlier et al., 2014; Lalor et al., 2021). Despite the increasing number of people suffering from helminthiasis, these diseases are still classified as ‘neglected’ by the World Health Organization (WHO), mainly due to the lack of resources destined to the investigation of these infections (Weatherhead et al., 2017).

Helminths establish long‐term infections by modulating the immune response in the host to promote their survival (Gazzinelli‐Guimaraes & Nutman, 2018; Maizels & McSorley, 2016; Ryan et al., 2020a; Sánchez‐López et al., 2021). *Dicrocoelium dendriticum* and *Fasciola hepatica* are two hepatic trematodes found worldwide than can coexist in the same host, complicating their diagnosis. They both live in the liver bile ducts of many mammalian species, including humans, but they have significant differences in their life cycle and pathology. Histopathological analysis after infection with *F. hepatica* or *D. dendriticum* shows the presence or the absence of migratory tracts in the liver parenchyma respectively, which suggests the possibility of a different immune response to these two trematodes (Piegari et al., 2021; Rakho, 1972; Siles‐Lucas et al., 2021). *F. hepatica* infections have been widely studied, confirming the extraordinary ability of this trematode to modulate the immune response throughout all developmental stages within the definitive host (Ryanet al., 2020a; Siles‐Lucas et al., 2021). In contrast, there are only a few reports of the immune response elicited by *D. dendriticum* infection (Ferreras‐Estrada et al., 2007; Piegari et al., 2021).

The modulation of the host response in helminthiases is mostly due to the release of molecules at the host‐parasite interface, commonly referred to as Excretory/Secretory Products (ESP), including some specific proteins, lipids, metabolites, nucleic acids, as well as Extracellular vesicles (EVs) (Maizels et al., 2018; Sánchez‐López et al., 2021). EVs are increasingly recognized as important mediators of cell‐cell communication. EVs are small membrane‐enclosed nanoparticles actively released by cells that are classified depending on their biogenesis, biophysical and biochemical properties (Buzas, 2022). Nonetheless, as current technology cannot discriminate between these subpopulations, and attending to the most recent guidelines from the International Society of Extracellular Vesicles (ISEV), the generic term ‘Extracellular Vesicles’ will be used here (Théry et al., 2018). EVs have a key role in intercellular communication and can carry diverse cargo, including bioactive molecules, surface receptors, and genetic information (such as non‐coding RNAs) (Maas et al., 2017; Pitt et al., 2016). EV secretion by helminths was first described in *F. hepatica* and *Echinostoma caproni* (Marcilla et al., 2012) and since then, numerous studies have highlighted their relevance in host‐parasite communication, through the modulation of the host immune responses (Drurey & Maizels, 2021; Hoffman et al., 2020; Mu et al., 2021; Sánchez‐López et al., 2021). Several studies have thoroughly investigated the biogenesis and content of *F. hepatica* EVs (*Fh*EVs) (Cwiklinski et al., 2015; Bennett et al., 2020; Davis et al., 2019; de la Torre‐Escudero et al., 2019; Fromm et al., 2015; Murphy et al., 2020; Ovchinnikov et al., 2020; Sánchez‐López et al., 2020) and their immunomodulatory properties (de la Torre‐Escudero et al., 2016; Roig et al., 2018; Murphy et al., 2020). In contrast, to our knowledge, there is only one report characterizing the protein and miRNA content of *D. dendriticum* EVs (Bernal et al., 2014), with no further reports analyzing their functional properties.

Interestingly, ESP, including EVs from different helminths, have been shown to have anti‐inflammatory properties, suggesting their possible use in treating allergic disorders and autoimmune diseases (Buck et al., 2014; Drurey & Maizels, 2021; Eichenberger et al., 2018; Roig et al., 2018; Ryan et al., 2020b). We have previously reported that EVs from *F. hepatica* were able to prevent Dextran Sodium Sulfate (DSS)‐induced colitis in C57BL/6 mice, as a model of inflammatory bowel disease (IBD) (Roig et al., 2018). *Fh*EVs produced a protective effect mediated by a significant downregulation of pro‐inflammatory cytokines, such as TNF‐α, IL‐6 and IL‐17a, suppression of MAPK/NF‐κβ signaling pathways, and reduced neutrophil infiltration. Interestingly, these effects did not depend on mature lymphocytes. Given that the role of dendritic cells (DCs) is typically focused on T cell polarization (Mellman, 2013), the most likely candidate cells to mediate this host‐parasite interaction are macrophages, which could phagocytose most of the EVs.

Macrophages are major regulators of the inflammatory response, categorized as M1 pro‐inflammatory macrophages, based on their production of inflammatory cytokines and anti‐microbial molecules or M2 anti‐inflammatory macrophages, with a marked tissue‐repair function (Yunna et al., 2020). Modulatory effects of helminth EVs have been reported in human and murine macrophages (Sánchez‐López et al., 2021) and they are the primary immune cells present during the first days of infection with *F. hepatica* (Ruiz‐Campillo et al., 2018). In addition, the presence of immunomodulatory miRNAs commonly found in *Fh*EVs, such as *fhe‐miR‐125b* (Fromm et al., 2015), have also been found in macrophages from infected animals (Tran et al., 2021). Furthermore, Espino and colleagues demonstrated the strong modulatory effect of the ESP from *F. hepatica* in the macrophage THP1‐XBlue™ CD14 cell line, where proteins such as Glutathione‐S‐Transferase (GST) or cathepsins, are powerful anti‐inflammatory molecules (Aguayo et al., 2019; Figueroa‐Santiago & Espino, 2017; Ramos‐Benítez et al., 2017). Importantly, these proteins have also been detected in *Fh*EVs (Cwiklinski et al., 2015).

The aim of the present study is to compare the protein cargo of the EVs from *F. hepatica* and *D. dendriticum* isolated by Size Exclusion Chromatography (SEC) and to study their effect on macrophages in culture. We show here an origin‐dependent effect of EVs.

## 2 MATERIALS AND METHODS

### 2.1 Helminth ESP collection

A total of 60 adults of *F. hepatica* and 600 adults of *D. dendriticum* (in 6 isolations) were obtained from infected cattle livers from local slaughterhouses. The collected parasites were washed thoroughly three times with PBS previously filtered using 0.22 μm filters (Thermo Fisher Scientific). Parasites were incubated at concentrations of two flukes/ml for *F. hepatica* and 10 flukes/ml for *D. dendriticum* for 4 h at 37°C in RPMI‐1640 with L‐glutamine (Gibco) containing 100 μg/ml streptomycin and 100 U/ml penicillin (all from Sigma‐Aldrich), in the presence of protease inhibitors (cOmplete™, EDTA‐free, Roche). After the incubation period, the parasite culture media (containing the ESP) was collected and centrifuged at low speed, first at 750 × g/15 min and then at 3000 × g/15 min, to remove larger debris. The supernatant (ESP) was collected and stored at −80°C until further processing.

### 2.2 EV isolation by SEC and protein quantification

The isolation of EVs from parasites was performed as described previously (Sánchez‐López et al., 2020) and following the recent recommendations issued by White et al. (2023). Briefly, ESP were centrifuged at 16,000 × g/20 min at 4°C and filtered through 0.22 μm membrane filters to exclude large EVs. Then, the supernatant was loaded onto Amicon^®^ Ultra‐4 Centrifugal Filters (EMD Millipore) and centrifuged at 3200 × g for 15 min at 4°C. This step was repeated with the same filter until reaching 500 μl of final volume. EVs were isolated by SEC using syringes containing 10 ml of stacked Sepharose‐CL2B (Sigma‐Aldrich), as described previously (Sánchez‐López et al., 2020). Concentrated samples were loaded into the columns, and 20 fractions of 0.5 ml were collected from each sample. EV‐enriched fractions (6‐10) were pooled and loaded onto an Amicon^®^ Ultra‐4 Centrifugal Filter, and centrifuged at 3200 × g/15 min at 4°C. Fraction 1 was collected and used as a negative control. EV‐depleted fractions (F17‐20 SEC) were also pooled and collected to perform further experiments. To isolate EVs from differentiated THP‐1‐XBlue™‐CD14 cells, 2 ml of cell culture supernatants were processed using syringes containing 1 ml of stacked Sepharose‐CL2B. 12 fractions of 75 μL were collected. Fractions 4–7 were pooled as the EV‐enriched fraction, and fractions 9–12 were pooled and selected as the EV‐depleted fraction (F9‐12SEC). To measure the protein concentration using Micro BCA protein assay kit (Thermo Fisher Scientific) the pooled fractions were dissolved in 0.1 % Triton X‐100. Absorbance was measured at 595 nm on an iMark™ Microplate Absorbance Reader (Bio‐Rad). To store the different EV samples at −80°C, sucrose (Sigma‐Aldrich) was added to a final concentration of 10%.

### 2.3 Nanoparticle tracking analysis (NTA)

The size distribution of EVs was determined by NTA in a NanoSight LM10 (Malvern Instrument Ltd, Malvern), using a 405 nm laser and a scientific Complementary metal‐oxide semiconductor (sCMOS) camera. Data were analyzed with the NTA software version 3.3 (Dev Build 3.3.104), with Min track Length, Max Jump Distance and Blur set to auto, and the detection threshold set to 5. The camera level was set to 15, and five readings of 30 s at 30 frames per second. Images were taken with manual monitoring of temperature. Samples were diluted with filtered PBS to reach the concentration (20‐120 particles/frame) recommended by the manufacturer.

### 2.4 Transmission electron microscopy (TEM)

Samples were processed as described previously (Théry et al., 2006) with several modifications. Briefly, 8 μl of EV‐enriched samples were fixed in 2% Paraformaldehyde (PFA) for 30 min, and deposited on Formvar‐carbon coated EM grids for 15 min. Then, samples were washed with PBS and post‐fixed with 1% Glutaraldehyde for 5 min, washed with distilled water, and then contrasted in a mixture of uranyl acetate (1%) and methyl cellulose (0,5%). Samples were analyzed either with a Jeol JEM1010 or a Hitachi HT7800 transmission electron microscope (Servicio Central de Soporte a la Investigación Experimental (SCSCIE), Universitat de València). JEM1010 was operated at 80 kV and images were recorded on a MegaView III digital camera. EV size was determined using the Olympus Image Analysis Software. Hitachi HT7800 was operated at 100 kV and images were recorded on a CMOS EMSIS XAROSA digital camera, and EV size was determined using the ImageJ software (Schneider et al., 2012).

### 2.5 Immuno‐gold staining

Immunogold labelling of EVs was carried out at the Prince Felipe Research Centre of Valencia, Spain (CIPF) using EV‐enriched samples fixed in 2 % Paraformaldehyde in PBS for 30 min, and carbon‐coated nickel grids. Grids containing the EVs were washed in PBS, and blocked with PBS/0.1 M glycine/ 0.3 % BSA for 10 min. Grids were then incubated for 1 h with rabbit sera containing polyclonal antibodies (1:50) raised against *F. hepatica* TSG101 and the external domains of tetraspanin‐CD63 receptor (CD63rec) as primary antibodies (de la Torre‐Escudero et al., 2019), kindly provided by Dr. Mark Robinson, Queen's University, Belfast. After a washing with blocking solution for 20 min, the grids were incubated for 1 h with the gold‐labelled secondary antibodies (1:20) goat anti‐rabbit coupled to 12 nm gold particles (Abcam). Samples incubated with secondary antibodies alone were used as a control. After washing with PBS/0.1 M glycine, the grids were negative stained as described above and then viewed using a Jeol JEM1010 or a Hitachi HT7800 transmission electron microscope as previously described.

### 2.6 THP1‐XBlue™ CD14 cells, culture conditions and differentiation into macrophages

THP1‐XBlue™ CD14 (InvivoGen) is a cell line derived from human monocytes that stably expresses a macrophage‐specific differentiation antigen (CD14) that interacts with several Toll‐Like Receptors (TLRs). This cell line is derived from the THP‐1 via transfection with a reporter construct expressing a secreted embryonic alkaline phosphatase (SEAP) gene under the control of a promoter that is inducible by the transcription factor NF‐kB and AP‐1. When these transcription factors are activated, the expression of NF‐kB and AP‐1 is induced, secreting SEAP. The cell response can be monitored using QUANTI‐Blue™, which facilitates monitoring of TLR‐induced NF‐kB/AP‐1 activation (Chanput et al., 2014). This method has been widely used for trematode ESP, as previously mentioned (Aguayo et al., 2019; Figueroa‐Santiago & Espino, 2017; Ramos‐Benítez et al., 2017). THP‐1‐XBlue™‐CD14 cells were grown and maintained in RPMI‐1640 culture medium supplemented with 10% of Fetal Bovine Serum (FBS), L‐glutamine (all from Gibco), 100 μg/ml streptomycin and 100 U/ml penicillin (all from Sigma‐Aldrich). Cells were grown in 75 cm^2^ flasks (Gibco) at 37°C, 5% CO_2_ and 100% humidity. To differentiate THP1‐XBlue™ CD14 cells into macrophages, cells were cultured to a concentration of 3 × 10^5^ cells/ml and stimulated with phorbol 12‐myristate 13‐acetate (PMA) for 72 h at a final concentration of 100 ng/ml. The culture medium from the differentiated macrophages was replaced with fresh medium and cells were cultured for another 72 h prior to EVs addition. The cell viability was quantified with Trypan blue (Gibco), performing a 1:1 mixture with the cells. Cells were counted in a Countess™ Cell Counting Chamber (Thermo Fisher Scientific).

### 2.7 NF‐κβ activation screening assay

The THP1‐XBlue™ CD14 cells contain an embryonic alkaline phosphatase (SEAP) reporter gene induced by NF‐κβ transcription factor, allowing for the measurement of its activation with the colorimetric reagent Quanti‐blue™ (InvivoGen). For the NF‐κβ activation screening assay, cells were differentiated and grown in 96‐well plates, then they were washed with 0.22 μm filtered PBS, and FBS‐free medium was added. One of the following treatments were then added to macrophages: PBS (10 μl), *Fh*EVs (5 and 10 μg/ml), *Dd*EVs (5 μg/ml), the SEC EV‐depleted fractions (F17‐20 *Fh*SEC; F17‐20 *Dd*SEC at concentrations ranging from 10 to 40 μg/ml), the first fraction (F1) derived from *Fh*EVs SEC purification (10 μl), or with lipopolysaccharide (LPS) from *Escherichia coli* K12 (Invivogen) to a final concentration of 300 ng/ml as a positive control of inflammation. Cells were incubated at 37°C, 5% CO_2_ for 24 h. In addition, to study the potential anti‐inflammatory properties of *Fh*EVs and *Dd*EVs on LPS‐activated macrophages, after 1 h of incubation with LPS (300 ng/ml), *Fh*EVs and/or *Dd*EVs were added to the samples. In all the experiments, after 24 h of incubation, 20 μl of each cell culture supernatant were mixed with 180 μl of Quanti‐blue™ reagent (InvivoGen) and incubated for 5 h at 37°C, following the manufacturer instructions. Absorbance was measured at 650 nm with a microplate reader (iMark™ Microplate Absorbance Reader, Bio‐Rad).

### 2.8 Enzyme‐linked immunosorbent assay (ELISA)

Maxisorp microtiter plates (Thermo Fisher Scientific) were coated overnight at 4°C with either 2.5 μg of total extracts of proteins from lysed cell pellets, with 0.05% Triton X‐100 (BioRad), or with 2.5 μg of each protein enriched‐fraction in 50 mM carbonate buffer (pH 9.6). The coated plates were washed 3 times with PBS, 0.05% Tween 20 (PBST) (BioRad) and uncoated sites were blocked with 5% BSA in PBST for 1 h at 37°C. Samples were incubated for 1.5 h at 37°C with 100 μl of either mouse α‐RAC1 (1:250) (Abcam), rabbit α‐Annexin A2 (1:1000) (Abcam), α‐PSMD6 (1:500) (Novus Biologicals), α‐IFIT3 (1:500) (Cell signalling Technology®) or α‐GAPDH polyclonal sera (1:500) (kindly provided by Dr. Daniel Gozalbo, Universitat de València). Then, plates were washed three times with PBST and incubated with 100 μl of goat anti‐rabbit or anti‐mouse coupled to horseradish peroxidase (Jackson Laboratories) as secondary antibodies, both diluted 1:10000 in PBST. After incubation for 1 h at 37°C, wells were washed three times with PBST and incubated with 50 μl of substrate solution (0.012% hydrogen peroxide, 0.4 mg/ml of ortho‐phenylenediamine (Sigma) in 0.05 phosphate‐citrate buffer, pH = 5.0), in darkness for 15 min at room temperature. The reaction was stopped with 50 μl of 3N HCl. Absorbance was measured at 490 nm in an iMark™ Microplate Absorbance Reader (Bio‐Rad). TNF‐α was measured using a commercially‐available kit (TNF alpha Human ELISA Kit (Thermo Fisher Scientific)), according to the manufacturer's instructions.

### 2.9 Quantification of cytokines mRNAs by RT‐qPCR

For RT‐qPCR assays, we followed a protocol previously described (Trelis et al., 2016), with several modifications. THP1‐XBlue™ CD14 cells were differentiated into macrophages and grown in 6‐well plates. Then they were washed with 0.22 μm filtered PBS, and FBS‐free medium was added. Macrophages were then treated with either PBS, *Fh*EVs (10 μg/ml) or *Dd*EVs (5 μg/ml) and cell pellets were obtained after 24 h of incubation. In addition, macrophages were stimulated with 300 ng/ml of LPS, and after 1 h of incubation cells were treated with either PBS or *Fh*EVs (10 μg/ml) and cell pellets were obtained after 24, 48, and 72 h post‐treatment. The total RNA from cells was purified with the RealTotal RNA spin plus Purification Kit (Real), according to manufacturer's guidelines, and the quality and quantity of RNA was assessed using a Nanodrop system (Thermo Fisher Scientific). The obtained RNA was reverse transcribed into cDNA using a High Capacity cDNA Reverse Transcription Kit (Applied Biosystems). The mRNA corresponding to CXCL8, TNF‐α, TGF‐β, IFN‐γ, IL‐1β, IL‐10, IL‐4, IL‐6, IL‐13, IL‐5, NOS2, RETN, ARG1, CD80, MRC1, and β‐actin were analyzed by qPCR, using the TaqMan™ Fast Advanced Master Mix and the primer pairs specific for every target (all designed by Applied Biosystems, Thermo Fisher Scientific). The StepOne™ real‐Time PCR System (Applied Biosystems) was used to perform the amplification reactions in 96‐well plates, using the following cycling conditions: initial setup of 2 min at 50°C, 10 min at 95°C, and 40 cycles of 15 s for denaturation at 95°C and 1 min of annealing at 60°C. On each plate, an endogenous control and a negative control were analyzed in duplicate. For RT‐PCR analysis, β‐actin was used as a reference gene for comparisons, and the relative amount of each mRNA was calculated using the comparative C_t_ method (ΔΔC_t_), with the final data derived from 2^−ΔΔCt^.

### 2.10 LC‐MS/MS analysis and database search

An automated sample preparation pipeline for low‐input proteomics based on the single‐pot solid‐phase‐enhanced sample preparation (SP3) method was used for the proteomic analysis of purified EVs and macrophages after the treatments (Müller et al., 2020). THP1‐XBlue™ CD14 cells were differentiated and grown in 6‐well plates and the culture media of three wells, after 24 h of incubation with each treatment, were used for each determination. Three replicates were used for each treatment. To avoid contaminants, we used a combination (1:1) of two different types of carboxylate‐modified magnetic particles (Thermo Scientific Sera‐Mag SpeedBeads™) as described previously (Trelis et al., 2022). Dried EV samples were dissolved in 30 μl of 50 mM ammonium bicarbonate (ABC) each. 4 μl of beads and acetonitrile (ACN) to a final concentration of 70% (v/v) were added to each sample. After incubating for 20 min at RT, the sample/bead mixture was placed on a magnetic rack for 2 min. After discarding the supernatant, the beads were washed and peptides digested as described (Trelis et al., 2022). Five microliters of the resulting mixtures (concentrated by speed vacuum to 10 μl) were analyzed by LC–MS/MS first, desalting the samples with 0.1% TFA at 5 μl/min during 5 min using a trap column (3 μm C18‐CL, 350 μm × 0.5 mm; Eksigent). The peptides were then loaded onto an analytical column (3 μm C18‐CL 120 Å, 0.075 × 150 mm; Eksigent) as described by Cwilinski et al. (2015), equilibrated in 5% acetonitrile, 0.1% formic acid (FA). The elution was carried out with a linear gradient of 15%–40% of solution B (ACN, 0.1% FA) in solution A (0.1% FA) for 60 min at a flow rate of 300 nl/min. Peptides were analyzed in a mass spectrometer nanoESI qQTOF (6600plus TripleTOF, ABSCIEX) at SCSIE, UV. Samples were ionized in a Source Type: Optiflow < 1 μl Nano applying 3.0 kV to the spray emitter at 175°C. Analysis was carried out in a data‐dependent mode. Survey MS1 scans were acquired from 350−1400 m/z for 250 ms. The quadrupole resolution was set to ‘LOW’ for MS2 experiments, which were acquired 100−1500 m/z for 25 ms in ‘high sensitivity’ mode. Protein identification was performed using the ProteinPilot v5.0. search engine, using the Paragon algorithm (Shilov et al., 2007) to search the Uniprot_Trematoda database for EVs containing samples, and Uniprot_Human database for differentiated THP1‐XBlue™ CD14 cells. For the label‐free quantitative proteomics, the obtained quantitative data for peptides were analyzed by Marker View 1.3 (Sciex). First, areas were normalized by total areas summa, and then a PCA, DA analysis and Student *t*‐test were performed. Protein‐protein interaction networks of the identified proteins were created using the STRING database with default parameters (Szklarczyk et al., 2019). The proteomic analysis was also performed in the proteomics facility of the SCSIE, UV.

### 2.11 Cell migration assays

1 × 10^6^ THP1‐XBlue™ CD14 monocytes were seeded into the upper compartment of a 12‐well plate with 3 μm pore Transwell inserts (Falcon®) in RPMI 1640 medium with 0.1% FBS. This culture medium was also added to the lower chamber of the Transwell plate. The cultured cells in the upper compartment were treated either with PBS (100 μl), *Fh*EVs, *Dd*EVs, the EV‐depleted fractions (F17‐20 for *Fh*SEC and F17‐20 for *DdSEC*) (all at 10 μg/ml), or with LPS from *E. coli* K12 (InvivoGen) to a final concentration of 300 ng/ml as a positive control of migration. Cells were incubated at 37°C, 5% CO_2_ for 48 h and then, the transwell insert was removed. Migrated cells were imaged using a Zeiss Primovert inverted microscope, with an Axiocam 202 mono camera (Zeiss). The number of cells that had migrated to the lower compartment were counted using a Countess™ Cell Counting Chamber (Thermo Fisher Scientific).

### 2.12 Statistical analysis

Data were expressed as the mean ± SEM. Statistical significance was determined with a one‐way analysis of variance (ANOVA). For parameters not showing normal distribution, we applied the Kruskal‐Wallis non‐parametric test. For comparisons between two groups, the Student *t*‐test was used. Differences in mean values were considered statistically significant when *p* < 0.05. Analysis and graphs were performed using GraphPad Prism 8 software (San Diego, CA, USA).

## 3 RESULTS

### 3.1 EVs from *F. hepatica* and *D. dendriticum* exhibit differences in their proteome content, but share common biological features

**FIGURE 1:**
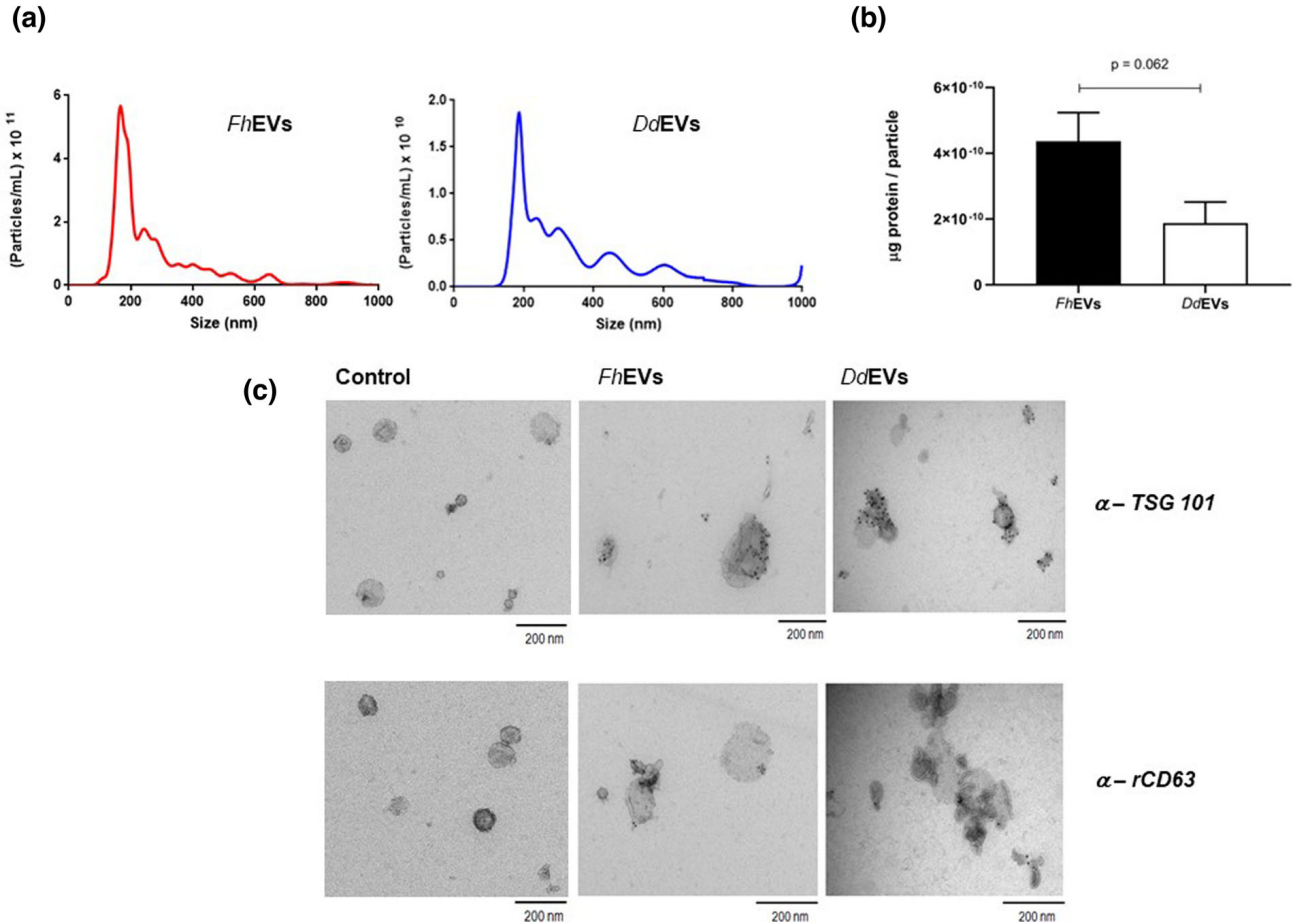
Characterization of Extracellular Vesicles obtained from *F. hepatica* and *D. dendriticum*. (a) *Fh*EVs and *Dd*EVs were successfully isolated by SEC and EV concentration and size distribution were measured by Nanoparticle Tracking Analysis. (b) The ratio between the μg of protein and the total particle number indicates the protein enrichment of the purified EVs. (c) TEM‐Immunogold labelling confirmed the presence of the typical trematode EVs (with large and small EVs) and their associated markers, TSG101 and rCD63. Control samples from *Fh*EVs incubated with the secondary antibody alone are shown on the left. One‐way ANOVA was performed as statistical analysis with Graph Pad Prism 8, *p < 0.05, **p < 0.01, ***p < 0.001.

We isolated EVs from *D. dendriticum* and *F. hepatica* using an approach based on differential ultracentrifugation, followed by SEC. To verify their presence and characterize the EVs (size and morphology), both NTA and TEM were used (EVtrack ID EV220006), as recommended by White et al. (2023). The isolated EVs were visualized by TEM, confirming the presence of typical round‐shaped vesicles within the size range of 30–200 nm (data not shown). However, whereas *Dd*EVs only displayed round‐shaped morphologies, some of them coated with electrodense spikes, *Fh*EVs showed a wide range of diverse morphologies, also including large or small tubules, as previously described (Sánchez‐López et al., 2020). Particle concentration estimation by NTA was 3.12 ± 1.53 × 10^11^ for *Fh*EVs and 8.36 ± 1.51 × 10^10^ particles/ml for *Dd*EVs, with an average modal size of 169.1 ± 10.5 nm and 181.7 ± 39.2 nm, for *Fh*EVs and *Dd*EVs, respectively (Figure 1a). Furthermore, the determination of the ratio between the protein concentration and the particle number showed that *Fh*EVs (4.37 ± 1.74 × 10^−10^ μg of protein/particle) were more enriched in protein than *Dd*EVs (1.88 ± 1.28 × 10^−10^ μg of protein/particle) (Figure 1b).

To further confirm the presence of typical EV‐associated markers in purified EVs, immunogold labelling and TEM were performed using specific antibodies against *F. hepatica* TSG101 and CD63rec (de la Torre‐Escudero et al., 2019). Both markers were localized on the surface of both *Dd*EVs and *Fh*EVs, but TGS101 was more abundant than rCD63. We did not observe any gold labeling in EVs incubated only with secondary gold‐labelled antibodies as a control (Figure 1c).

To compare the protein cargo of *Fh*EVs and *Dd*EVs, their protein content was analyzed by LC‐MS/MS, and proteins were identified using the Uniprot/SwissProt database (https://www.uniprot.org). Protein identifications were established when values were greater than 95.0% probability, and contained at least 2 identified peptides for *Fh*EVs proteins, and 1 peptide for *Dd*EVs proteins. We used these criteria since there are very few reports on the proteomics of *D. dendriticum* ESP (Bernal et al., 2014; Martínez‐Ibeas et al., 2013) and data from *D. dendriticum* in NCBI or Uniprot/SwissProt are scarce. With these standards, 238 proteins in *Fh*EVs and 87 proteins in *Dd*EVs were identified (Supplementary Tables S1 and S2).

**FIGURE 2:**
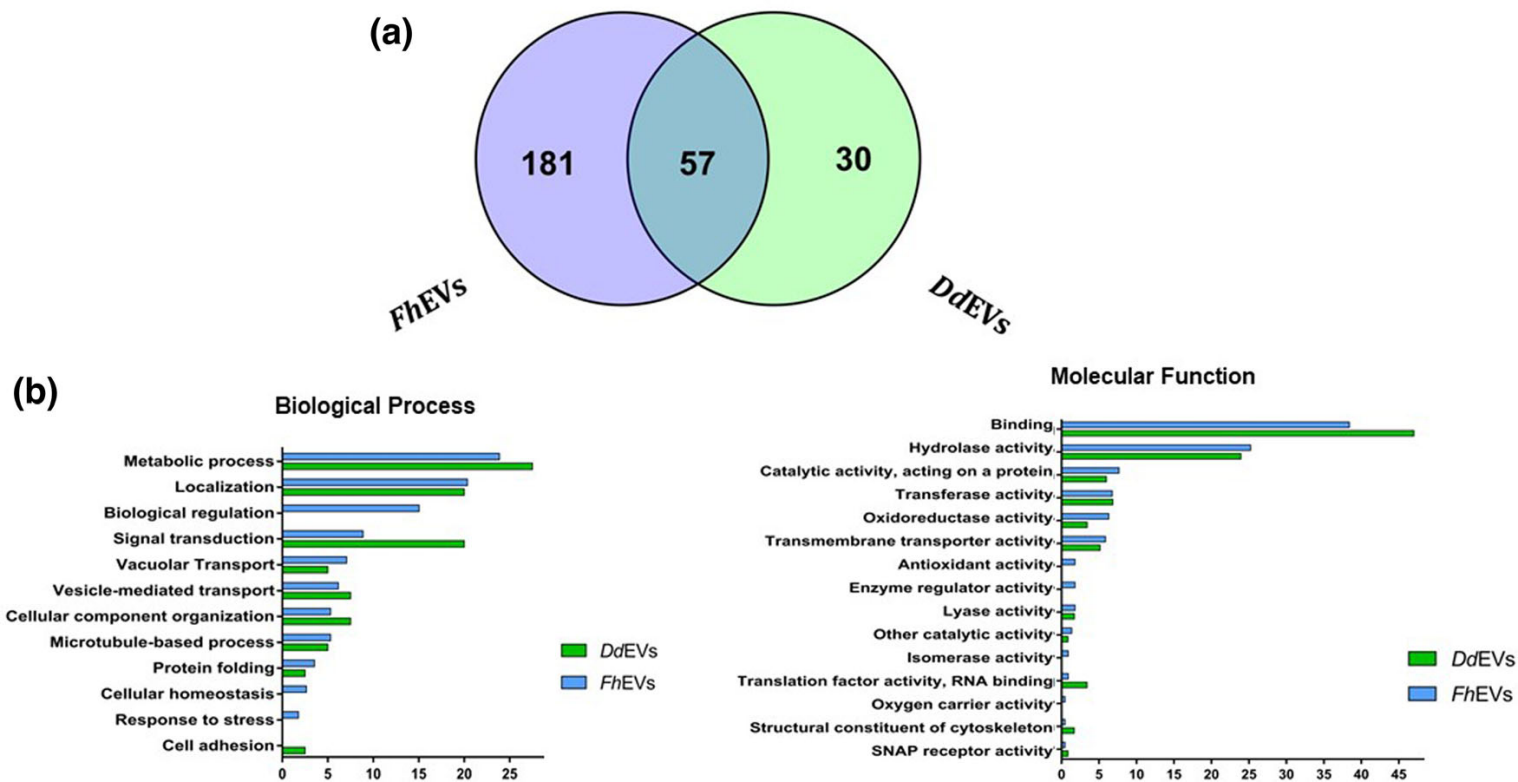
Characterization of the protein content of *Fh*EVs and *Dd*EVs. The proteome of *Fh*EVs and *Dd*EVs were analyzed by LC‐MS/MS. (a) The Venn diagram indicates the shared and unique proteins identified in *Fh*EVs and *Dd*EVs. (b) The number of identified proteins in the proteome of *F. hepatica* and *D. dendriticum* EVs distributed according to the biological process and molecular functions by GO terms.

EVs purified from *D. dendriticum* and *F. hepatica* presented 57 common proteins (Figure 2a), including Alix, several Rab proteins, heat shock proteins, annexins, cubilin and multivesicular body proteins, which are commonly identified in EVs (Montaño et al., 2021). Other commonly‐identified proteins include metabolic enzymes like GAPDH, calcium ion sensors like otoferlin or calmodulin, or proteins involved in signaling, like RAC1 (Supplementary Tables S1 and S2).

Gene Ontology (GO) analysis, using the PANTHER‐GO system (Version 16.0; http://www.pantherdb.org/), classified the identified proteins in two broad categories: 'Biological process' and 'Molecular Function' (Figure 2b). The five most enriched GO terms within the 'Molecular Function' ontology were 'Binding', 'Hydrolase activity', 'Catalytic activity, acting on a protein', 'Transferase activity' and 'Oxidoreductase activity'. Regarding the 'Biological process' ontology, the five most enriched GO terms were 'Metabolic process', 'Localization', 'Biological regulation', 'Signal transduction', and 'Vacuolar transport'.

### 3.2 *Dd*EVs and *Fh*EVs trigger different responses in human macrophages

**FIGURE 3:**
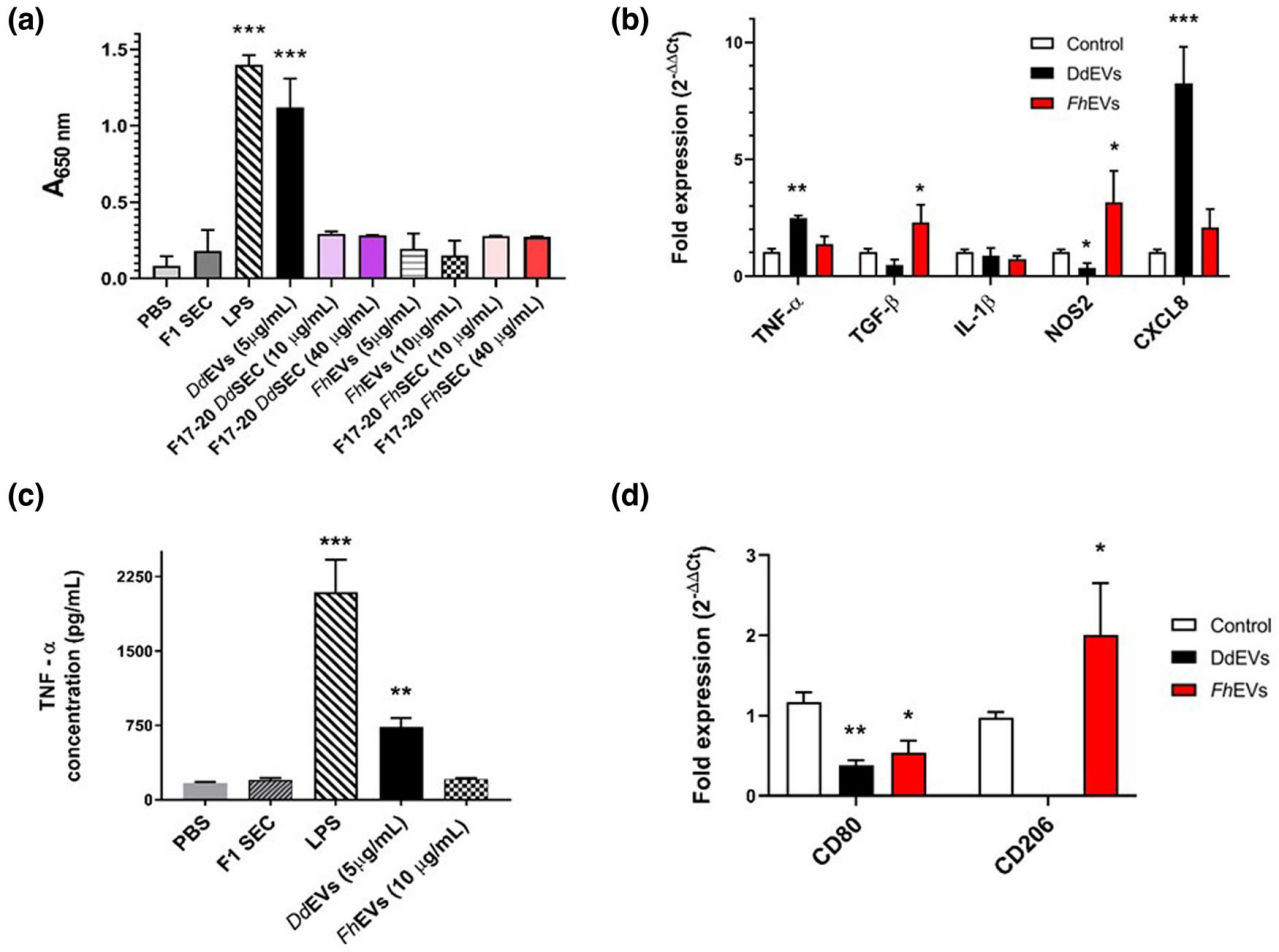
*Fh*EVs and *Dd*EVs induce a distinct phenotype in human macrophages in culture. Differentiated THP1‐XBlue™ CD14 cells were incubated with (a) *Dd*EVs 5 μg/ml and *Fh*EVs; or their EV‐depleted fractions from SEC (F17‐20 *Fh*SEC; F17‐20 *Dd*SEC), for 24 h, and NF‐κβ expression was measured in the supernatant using QUANTI‐Blue™. PBS and the fraction 1 eluted from the SEC column during *Fh*EVs isolation (F1 SEC) were used as negative controls, and LPS as a positive control of inflammation. PBS was used as negative control for statistical analysis. (b) Quantification by RT‐qPCR of several cytokines and chemokines in cells treated with PBS as control, *Dd*EVs (5 μg/ml) or *Fh*EVs (10 μg/ml). (c) TNF‐α concentration in the supernatants of macrophages determined by ELISA. (d) CD80 as a markers of macrophages M1 and CD206 as a marker of M2 polarization were measured by RT‐qPCR. Data are shown as mean ± SEM from 13 independent experiments for NF‐κβ expression assessment, five independent experiments for RT‐qPCR analyses, and from eight independent experiments for ELISA. One‐way ANOVA was performed as statistical analysis with Graph Pad Prism 8, *p < 0.05, **p < 0.01, ***p < 0.001.

Macrophages play an important role in trematode infections and they are the primary immune cells present during the first days of infection with *F. hepatica* (Ruiz‐Campillo et al., 2018). To investigate the response of macrophages to the addition of *Fh*EVs or *Dd*EVs, we analyzed the production of NF‐κβ 24 h after their administration to the differentiated THP1‐XBlue™ CD14 cultured cells. This human cell line has been widely used in similar studies as indicated above, and is widely used in cellular inflammation studies with similar functionality to human monocytes/macrophages. This cell line has several advantages, including: i) a more homogenous genetic background; ii) the ability to grow in unlimited passages providing high yield of cells; and iii) the ability to be transfected with non‐viral systems (Mohd Yasin et al., 2022). We used two concentrations of EVs as previously described (Trelis et al., 2016). In pilot studies, similar results were obtained using either 5 and 10 μg/ml of *Dd*EVs (data not shown) and in order to optimize the use of *Dd*EVs, the lower amount was used throughout the studies. The treatment with 5 μg/ml of *Dd*EVs induced an increase in NF‐κβ expression, similar to the increase produced by the administration of LPS (Figure 3a). In contrast, treatment with *Fh*EVs did not produce any significant variation on NF‐κβ expression, using either 5 μg/ml or 10 μg/ml of EVs (Figure 3a). The treatment with EV‐depleted fractions (F17‐20 SEC) from either *F. hepatica* or *D. dendriticum*, in concentrations up to 40 μg/ml, did not produce any effect, which confirms the specificity of *Dd*EVs in the immune modulation of macrophages (Figure 3a).

Based on these findings, we next screened the effects of the EVs on a panel of 11 different pro‐ and anti‐inflammatory cytokines and chemokines produced by macrophages (CXCL8, TNF‐α, TGF‐β, IFN‐γ, IL‐1β, IL‐10, IL‐4, IL‐6, IL‐13, IL‐5 and NOS2). The mRNA expression of these genes was evaluated by RT‐qPCR in cells after 24 h of exposure to *Dd*EVs or *Fh*EVs and compared to untreated macrophages (PBS). Differences in macrophage responses depending on the parasitic source of EVs were detected (Figure 3b). The exposure to *Dd*EVs increased TNF‐α and CXCL8 mRNA levels and decreased NOS2 levels. However, macrophages exposed to *Fh*EVs showed increased TGF‐β and NOS2 mRNA levels, with no significant variations observed for TNF‐α or CXCL8 mRNAs (Figure 3b). IL‐1β mRNA levels did not change with any of the treatments. No differences in the mRNAs corresponding to IFN‐γ, IL‐10, IL‐4, IL‐6, IL‐13, IL‐5, mRNAs were detected in macrophages under these conditions (data not shown). Furthermore, we investigated the levels of the cytokines TNF‐α and IL‐10 in THP‐1 cell culture supernatants by ELISA. Accordingly, TNF‐α levels were increased (Figure 3c), but IL‐10 was not detected (data not shown) after the treatment with *Dd*EVs, confirming the differences previously identified using RT‐qPCR.

We next investigated whether THP‐1 macrophages exposed to *Dd*EVs or *Fh*EVs present alterations in the production of at least one of the typical markers of M1 (CD80) or M2 (CD206) macrophage polarization. Addition of EVs from both trematodes decreased mRNA levels of CD80 in THP‐1 cells, but only *Fh*EVs increased mRNA levels of CD206. This marker was not detected in *Dd*EVs‐treated cells, suggesting its downregulation (Figure 3d). Future assays will confirm the protein expression of these and other molecules.

### 3.3 *Fh*EVs, but not *Dd*EVs, exert anti‐inflammatory effects in LPS‐activated macrophages

**FIGURE 4:**
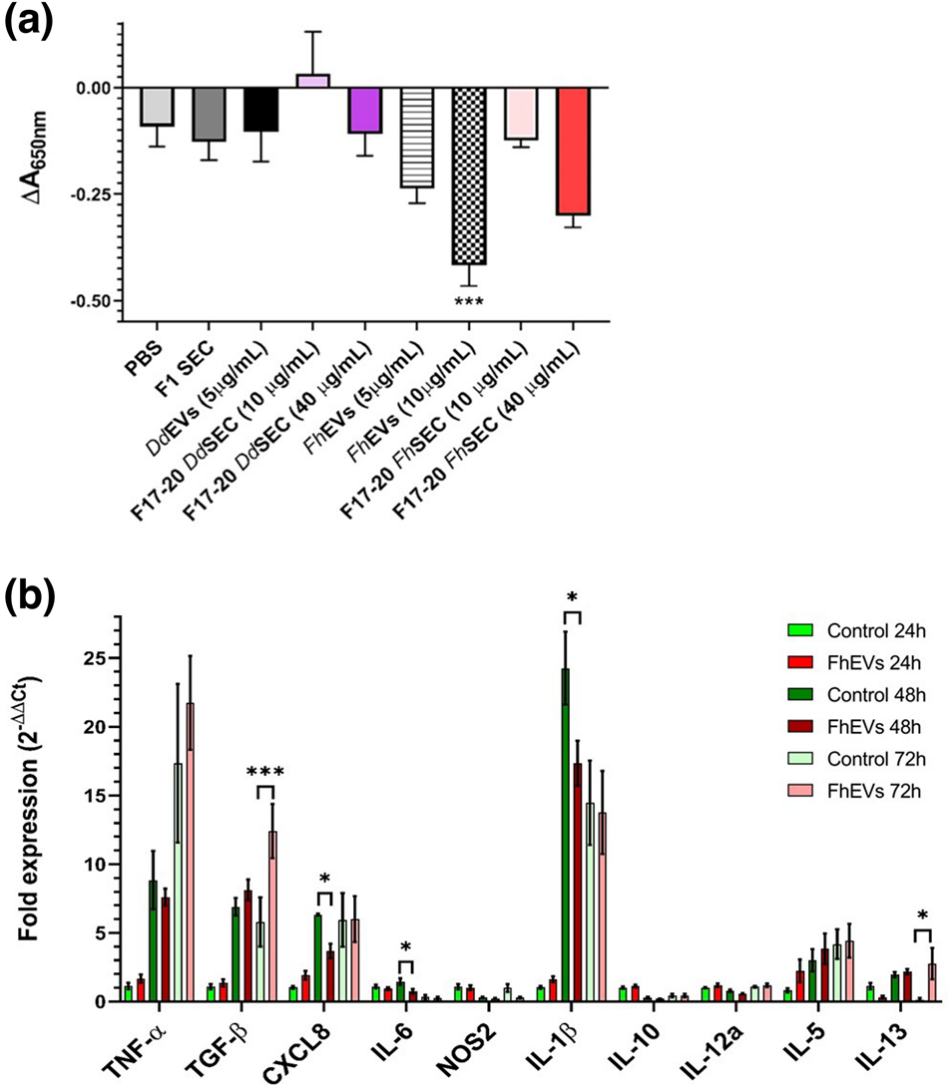
*Fh*EVs, but not *Dd*EVs, exert anti‐inflammatory effects on LPS‐activated macrophages. Differentiated THP1‐XBlue™ CD14 cells were stimulated with 300 ng/ml of LPS, and then treated with (a) *Fh*EVs and *Dd*EVs, or their EV‐depleted fractions from SEC (F17‐20 *Fh*SEC; F17‐20 *Dd*SEC) for 24 h. NF‐κβ expression was measured in the supernatant using QUANTI‐Blue™. The results are expressed as the difference of A_650nm_ obtained after each treatment and the A_650nm_ obtained in samples stimulated with only LPS. PBS and the fraction 1 eluted from the SEC column during *Fh*EV isolation (F1 SEC) were used as negative controls. PBS was used as control for statistical analysis. (b) cDNA was obtained from cells at 24, 48, and 72 h post treatment with *Fh*EVs (10 μg/ml), and several cytokines and chemokines were analyzed by RT‐qPCR. Data are shown as mean ± SEM from 13 independent experiments for NF‐κβ expression assessment, and 3 independent experiments for RT‐qPCR analyses. One‐way ANOVA was performed as statistical analysis with Graph Pad Prism 8, *p < 0.05, **p < 0.01, ***p < 0.001.

We have previously described the potential therapeutic role of *Fh*EVs in a model of DSS‐induced colitis, suggesting the anti‐inflammatory properties of *Fh*EVs (Roig et al., 2018). To investigate whether macrophages could participate in this modulation, we analyzed the effect of *Fh*EVs or *Dd*EVs on differentiated THP1‐XBlue™ CD14 cells pretreated with LPS for 1 h, determining the production of NF‐κβ 24 h after EVs administration. *Fh*EVs, at 10 μg/ml, induced a significant inhibition of NF‐κβ expression, compared to negative controls (PBS or Fraction 1 derived from SEC) (Figure 4a), while *Dd*EVs did not promote any significant variation in NF‐κβ levels when added after LPS stimulation (Figure 4a). To confirm the specificity of *Fh*EVs, the treatment with EV‐depleted fractions (F17‐20 SEC) from either *F. hepatica* or *D. dendriticum*, in concentrations up to 40 μg/ml, showed no any anti‐inflammatory effect (Figure 4a).

To check whether this inhibition of NF‐κβ expression produced by *Fh*EVs could impact the cytokine expression profile of LPS‐activated macrophages, the same panel of pro‐ and anti‐inflammatory cytokines and chemokines (CXCL8, TNF‐α, TGF‐β, IFN‐γ, IL‐1β, IL‐10, IL12a, IL‐4, IL‐5, IL‐6, IL‐13, and NOS2) was selected to evaluate the effect of *Fh*EVs, at 10 μg/ml, on macrophages previously stimulated with LPS. Our results did not show any significant variation in the mRNA expression profile after 24 h exposure to *Fh*EVs (Figure 4b). Moreover, secreted IL‐10 and TNF‐α were analyzed by ELISA in THP‐1 cell culture supernatants, confirming that both cytokines did not show differences in their secretion 24 h after addition of *Fh*EVs (data non shown). Since the changes in NF‐κβ expression occurs prior to the production of different inflammatory cytokines, we also performed a time‐course experiment where the mRNA expression profile was analyzed after longer *Fh*EV treatments (48 and 72 h) (Figure 4b). The incubation of macrophages with LPS for 48 and 72 h, promoted an increase in the expression of TNF‐α, CXCL8, IL‐1β and TGF‐β, as well as a decrease on the expression levels of IL‐10 (Figure 4b). Interestingly, *Fh*EVs (10 μg/ml), in the presence of LPS, increased the levels of TGF‐β and IL‐13 mRNAs, as well as reduced the IL‐6, IL‐1β and CXCL8 expression. IFN‐γ mRNA was not detected at any time point (data not shown).

### 3.4 Quantitative proteomics reveal rapid protein variations in the macrophage proteome upon *Fh*EV exposure

**FIGURE 5:**
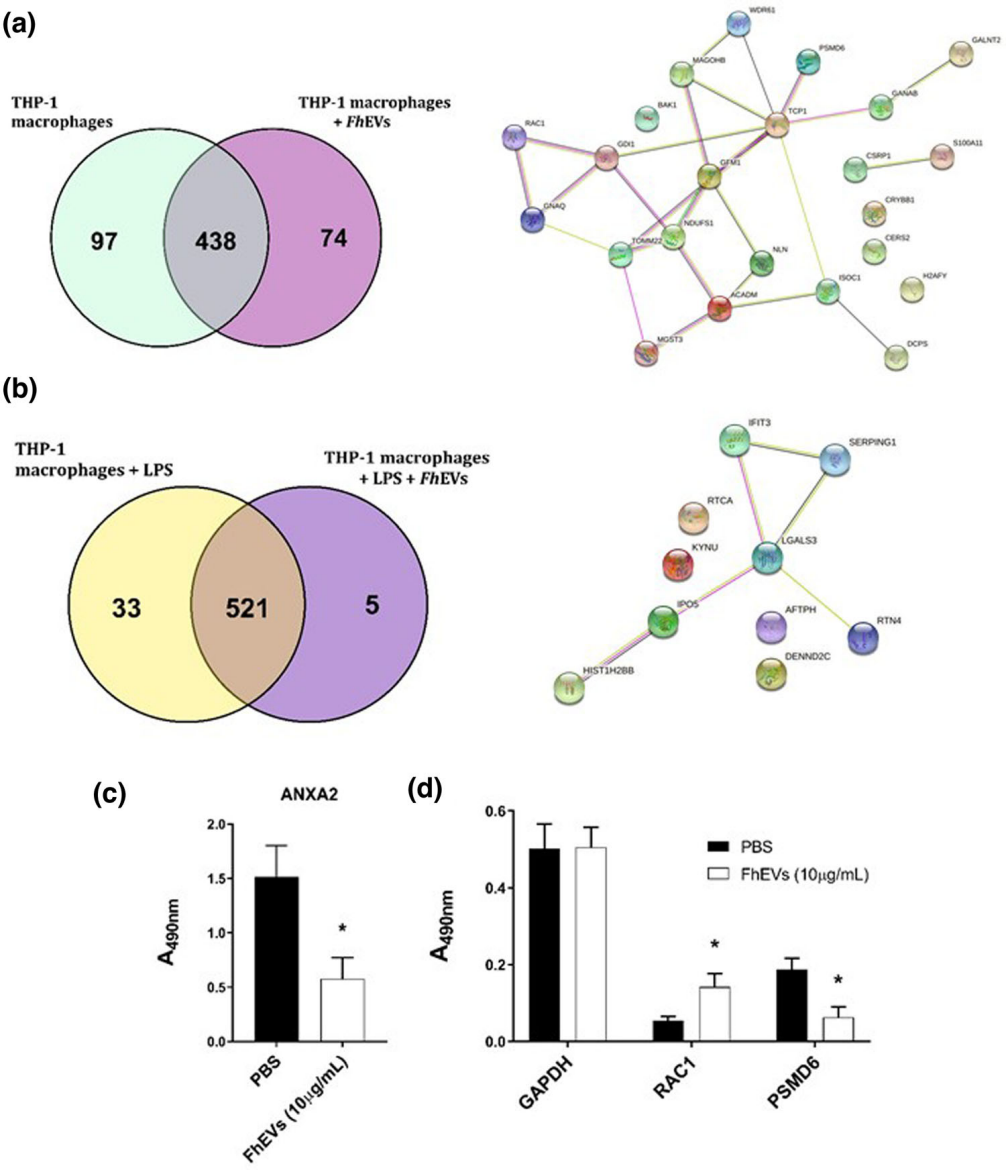
*Fh*EVs induce changes in the proteome of differentiated THP1‐XBlue™ CD14 cells. Macrophages were incubated with *Fh*EVs for 24 h, and cells and soluble protein fractions were analyzed by Label‐free quantitative proteomics. The Venn diagram of shared and unique proteins identified in the proteome of (a) cells treated with *Fh*EVs, and (b) cells stimulated with LPS and treated with *Fh*EVs, is represented along with the protein‐protein interaction network of the proteins with significant differential expression generated by the STRING database, using the minimum required interaction score (0.150). ELISA experiments to validate the proteomic results for (c) Annexin A2 (ANXA2) protein present in EV‐depleted fractions and (d) RAC1, PSMD6 as macrophage proteins. GAPDH was used as a control. Data are shown as the mean ± SEM from five independent experiments. One‐way ANOVA was performed as statistical analysis with Graph Pad Prism8, *p < 0.05, **p < 0.01, ***p < 0.001.

To study possible changes at the protein level in macrophages, we analyzed the number of proteins found in the proteome of macrophages 24 h after *Fh*EV treatment of either non‐stimulated and LPS‐stimulated cells. Protein identifications were established when values were greater than 95.0% probability, contained at least 2 identified peptides and was present in at least two out of three replicates. In non‐stimulated macrophages a total of 609 proteins were identified, from which 97 were only identified in non‐treated macrophages, and 74 in non‐stimulated macrophages treated with *Fh*EVs (Figure 5 and Supplementary Table 3). Upon *Fh*EV treatment of these non‐stimulated cells, 16 of the identified proteins, such as BAK and RAC1, were up‐regulated and 6 proteins, such as PSMD6, were down‐regulated (Table 1). On the other hand, 559 proteins were identified in LPS‐stimulated macrophages using the criteria described above (Supplementary Table 3). Upon treatment with *Fh*EVs, six proteins were up‐regulated and five down‐regulated (Table 1). To further characterize the early response of macrophages to parasite EVs, the analysis of the proteins secreted by the macrophages was carried out. Most of the EVs present in the cell culture supernatant were from parasite origin (data not shown). Therefore, we analyzed the soluble proteins, specifically the proteins present in the EV‐depleted fractions. In the assays without LPS, 192 proteins were identified, three of them increased upon *Fh*EV treatment (Table 1). In contrast, from the 201 proteins identified in LPS‐stimulated samples, only annexin A2 (ANXA2) protein levels were decreased upon *Fh*EV treatment (Table 1). To gain a functional assessment of the potential protein interactions of *Fh*EV targets, the proteomic data obtained for macrophages was analyzed with STRING (Figure 5). No significant enrichments were detected upon LPS‐stimulation and *Fh*EVs treatment, while the treatment with *Fh*EVs in the absence of LPS produced a functional enrichment related to tissue expression, such as ‘Liver’ and ‘Digestive gland’, which correlates with the parasite life cycle in the definitive host.

**TABLE 1:** List of the significant differentially expressed proteins in THP1‐XBlue CD14 cells upon *Fh*EVs treatment

	**Name**	**Accession number**	***p*‐Value**	**Fold change *Fh*EVs/control**
**No LPS—cells**	Bcl‐2 homologous antagonist/killer (Apoptosis regulator BAK) (Bcl‐2‐like protein 7)	Q16611	0.03736	6.45
	Cysteine and glycine‐rich protein 1 (Cysteine‐rich protein 1)	P21291	0.02481	4.44
	Protein mago nashi homolog 2	Q96A72	0.02931	3.99
	Histidine triad nucleotide‐binding protein 5	Q96C86	0.01935	3.87
	Ras‐related C3 botulinum toxin substrate 1 (RAC1)	P63000	0.0098	3.41
	Polypeptide N‐acetylgalactosaminyltransferase 2	Q10471	0.04119	2.90
	Guanine nucleotide‐binding protein G(q) subunit alpha	P50148	0.01958	2.88
	Alpha‐glucosidase 2	Q14697	0.045	2.57
	Core histone macro‐H2A.1	O75367	0.04287	2.55
	WD repeat‐containing protein 61	Q9GZS3	0.0415	2.42
	Elongation factor G, mitochondrial (EF‐Gmt)	Q96RP9	0.0382	2.37
	Beta‐crystallin B1	P53674	0.0421	2.32
	Isochorismatase domain‐containing protein 1	Q96CN7	0.00168	2.27
	NADH‐ubiquinone oxidoreductase 75 kDa subunit, mitochondrial (EC 7.1.1.2) (Complex I‐75 kD) (CI‐75 kD)	P28331	0.01995	2.14
	Angiotensin‐binding protein	Q9BYT8	0.03239	1.90
	S100 calcium‐binding protein A11	P31949	0,03536	1.58
	Medium‐chain specific acyl‐CoA dehydrogenase, mitochondrial (MCAD)	P11310	0.03119	0.66
	T‐complex protein 1 subunit alpha (TCP‐1‐alpha)	P17987	0.03156	0.48
	Microsomal glutathione S‐transferase 3	O14880	0.02118	0.34
	26S proteasome non‐ATPase regulatory subunit 6 (PSMD6)	Q15008	0.03545	0.34
	Mitochondrial import receptor subunit TOM22 homolog (hTom22)	Q9NS69	0.04591	0.31
	Ceramide synthase 2 (CerS2)	Q96G23	0.01098	0.17
**LPS—cells**	Immunoglobulin kappa variable 2D‐28	P01615	0.02142	4.22
	Plasma protease C1 inhibitor (SerpinG1)	P05155	0.02071	1.89
	Galectin‐3 (Gal‐3)	P17931	0.04902	1.75
	L‐kynurenine hydrolase	Q16719	0.03185	1.72
	Reticulon‐4	Q9NQC3	0.01126	1.59
	RNA 3′‐terminal phosphate cyclase	O00442	0.02756	1.34
	Aftiphilin	Q6ULP2	0.04398	0.44
	Interferon‐induced protein with tetratricopeptide repeats 3 (IFIT‐3)	O14879	0.04764	0.39
	Histone H2B type 1‐B	P33778	0.04084	0.37
	Importin‐5	O00410	0.04478	0.37
	DENN domain‐containing protein 2C	Q68D51	0.02977	0.31
**No LPS—F17‐20**	Calmodulin‐3	P0DP25	0.04835	3.88
	Keratin, type II cytoeskeletal 6A	P02538	0.0289	2.41
	Neuropilin‐1	O14786	0.01763	2.37
**LPS—F17‐20**	Annexin A2	P07355	0.03545	2.92

To further validate the proteomic results, we performed ELISA assays using antibodies against annexin A2, PSMD6 and RAC1, as well as with anti‐GAPDH as a control (Figure 5c,d). Our results confirmed that *Fh*EV treatment decreased annexin A2 levels in macrophages stimulated with LPS. Moreover, the ELISA experiments also validated the previously detected differences by proteomic analyses observed for PSMD6 and RAC1 (Figure 5).

### 3.5 *Fh*EVs, but not *Dd*EVs, reduce the migration of THP‐1 monocytes

**FIGURE 6:**
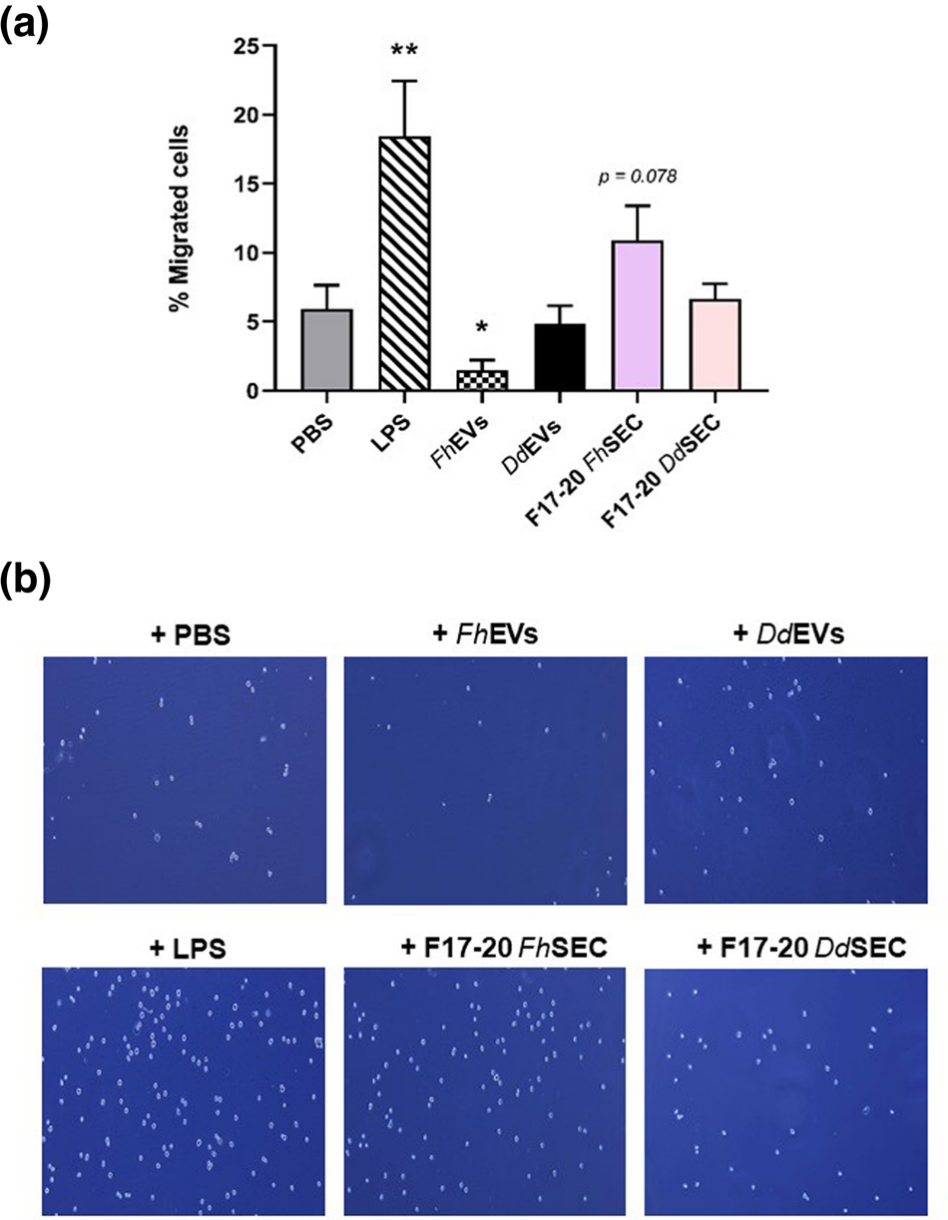
*Fh*EVs, but not *Dd*EVs, reduce the migration activity of THP‐1 monocytes. THP‐1 monocytes (1 × 10^6^ per well) were seeded in the upper chamber of 12‐well Transwell inserts, and migration assays were performed. Cells were treated either with *Fh*EVs, *Dd*EVs, or their SEC EV‐depleted fractions (F17‐20 *Fh*SEC; F17‐20 *Dd*SEC), all at 10 μg/ml, for 48 h. PBS was used as vehicle control, and LPS (300 ng/ml) was used as positive control for migration. (a) Results are expressed as percentage of cells that migrated from the upper chamber to the lower chamber of the Transwell, compared to the total cell number seeded. (b) Representative images obtained from the migration assays (100× magnification). Data are shown as the mean ± SEM of four independent experiments. One‐way ANOVA was performed as statistical analysis with Graph Pad Prism 8, *p < 0.05, **p < 0.01, ***p < 0.001 vs PBS.

As circulating monocytes are crucial in inflammatory processes after migrating to damaged or infected areas, we next tested whether *Fh*EVs or *Dd*EVs could modulate the migration of these cells in culture. For this purpose, LPS (300 ng/mL), *Fh*EVs, *Dd*EVs or fractions F17‐20 SEC derived from *F. hepatica* or *D. dendriticum* ESP (all at 10 μg/ml) were added to THP‐1 monocytes, and their migration capacity was evaluated using a transwell system 48 h after each treatment. The *Fh*EV treatment attenuated monocyte migration. In contrast, addition of fractions F17‐20 SEC derived from *F. hepatica* ESP induced a slight increase in monocyte migration, compared to the migration induced by LPS, used as the positive control (Figure 6). Neither EVs or F17‐20 SEC fractions derived from *D. dendriticum*, promoted any significant variation compared to control (PBS).

## 4 DISCUSSION

An increasing number of studies have reported the isolation and characterization of EVs from different helminths and describe their key role in host‐parasite communication, as well as describing their functional properties and therapeutic potential (Drurey & Maizels, 2021; Hoffman et al., 2020; Sánchez‐López et al., 2021). To study the effect of helminth EVs on macrophages, depending on their origin and composition, we have used EVs isolated by SEC from the closely related parasitic trematodes, *F. hepatica* and *D. dendriticum*, which exhibit differences in morphology and composition with sizes ranging from 30 to 200 nm, as described previously (see Drurey & Maizels, 2021; Sánchez‐López et al., 2021; and Tritten & Geary, 2018 for comprehensive reviews).

There are several reports on the proteomics of *Fh*EVs (Cwiklinski et al., 2015; Bennett et al., 2020; Davis et al., 2019; de la Torre‐Escudero et al., 2019; Murphy et al., 2020). Almost 90% of the proteins identified in the present study have been reported previously by Davis et al. (2019) which, as far as we know, is the only study that used SEC as the isolation method, confirming the high degree of reproducibility of this method. In our study, the implementation of a previous step of centrifugation at 16,000 × g and a filtration through a 0.22 μm membrane depleted our samples of many proteins, including proteasome subunits, detected in large EVs (Davis et al., 2019). In addition, the different search software used for protein identification (ProteinPilotv5.0. search engine) may also account for these differences, as has been previously described (Shteynberg et al., 2013). The only available proteomic analysis of *Dd*EVs used EVs isolated by ultracentrifugation (Bernal et al., 2014). Here, we report for the first time, the presence of Alix, as well as several Rab proteins in *Dd*EVs. Proteins from the Ras family are highly represented in EVs from helminths, and until now, they had not been identified in *D. dendriticum* (Bernal et al., 2014; Montaño et al., 2021).

The proteomic analyses of EVs from *F. hepatica* and *D. dendriticum* showed quantitative and qualitative differences. A higher number of proteins were identified in *Fh*EVs, as compared to *Dd*EVs, probably due to the absence of specific datasets for *D. dendriticum* proteins, which it is a critical point in proteomics when working with non‐model organisms (Heck & Neely, 2020; Toledo et al., 2011). Supporting this notion, EV protein markers such as TSG101 and CD63rec were detected using immuno‐gold labeling on both *Fh*EVs and *Dd*EVs, although proteomic analyses only identified these proteins in *Fh*EVs. Moreover, the EV protein marker Alix, as well as several Rab and heat shock proteins were also identified in EVs from both organisms, supporting the correct isolation of EVs. More than half of the proteins identified in *Dd*EVs were also present in *Fh*EVs, indicating a high degree of homology within the protein content of EVs in both trematodes, probably due to the presence of many similar structural proteins and enzymes participating in EV biogenesis (Cwiklinski et al., 2015). However, the 30 and 181 proteins identified only in *Dd*EVs or *Fh*EVs respectively, could be responsible for the different roles of these EVs. In addition, the differences observed in the functional classification of both proteomes show the absence of proteins within the GO categories 'Antioxidant activity' or 'Enzyme regulator activity' in *Dd*EVs.

Antioxidant systems in parasitic helminths have been proposed as defence mechanisms against the host‐generated oxygen radicals (Chiumento & Burschi, 2009). For instance, the immunomodulatory role of enzymes like thioredoxin (Dorey et al., 2021), identified in the proteomic analysis of *Fh*EVs, has been reported. Regarding the GO term 'Enzyme regulator activity', we highlight the absence of serpins (serine protease inhibitor) in *Dd*EVs. Previous reports have hypothesized their role in limiting the activation of the host immune response (Molehin et al., 2012) and they could account for the different phenotypic effect of the EVs on macrophages. A recent study has shown the depletion of the classical pathway of the complement system in response to *F. hepatica* infection, and its association with the expression of serine proteases by the parasite (De Marco Verissimo et al., 2022). In addition, our results show that *Fh*EVs up‐regulate the production of the SerpinG1 protein in LPS‐activated macrophages. It is likely that the combination of serine protease inhibitors secreted by the parasite (and identified in *Fh*EVs) and by the macrophages after internalizing *F. hepatica* ESP, ultimately lead to the inhibition of the complement system, and therefore, decreased inflammation. Further studies are needed to address the differences between both EV proteomes.

Helminth ESP, including EVs, have been under investigation as potential treatments for allergic disorders and autoimmune diseases (Buck et al., 2014; Coakley et al., 2017; Drurey & Maizels, 2021; Eichenberger et al., 2018; Roig et al., 2018; Ryan et al., 2020a). ESP from parasitic helminths target several cell types in order to elicit their characteristic immune regulatory phenotype, mainly affecting antigen‐presenting cells, such as DCs or macrophages (Ryanet al., 2020b). Murphy and co‐workers reported that *Fh*EVs modulate bone‐marrow derived DCs, enhancing the secretion of TNF‐α and the expression of cell surface (CD80, CD86, CD40, OX40L) and intracellular (SOCS1, SOCS3) co‐stimulatory markers (Murphy et al., 2020). However, the effects on DCs are not sufficient to explain all the immunomodulatory properties exhibited by this parasite during infection (Murphy et al., 2020). Macrophages are the main phagocytic cells that could take up the majority of the circulating EVs during the infection, suggesting a more important potential role for macrophages, rather than DCs, in the host response to *Fh*EVs. In this context, we have previously reported that *Fh*EVs prevent DSS‐induced colitis in mouse models, even in the absence of mature lymphocytes, suggesting that macrophages may be key players in helminth *Fh*EV immunoregulatory properties (Roig et al., 2018).

In the present study, we have investigated whether helminth EVs elicit different responses in cultured macrophages depending on their origin and composition. After a 24 h exposure to *Dd*EVs, macrophages showed increased expression of the transcription factor NF‐κβ, as well as of the cytokines TNF‐α and CXCL8, with a decrease in NOS2 mRNA levels. In contrast, NF‐κβ expression is not affected after the treatment with *Fh*EVs, and TGF‐β and NOS2 levels are increased. NF‐κβ is a transcription factor that regulates multiple cellular processes, including the production of pro‐inflammatory cytokines, chemokines and additional inflammatory mediators. In fact, NF‐κβ participates in the production of TNF‐α, IL‐1β, IL‐6 and CXCL8 (Liu et al., 2017). The differences observed in NF‐κβ expression upon treatment with *Fh*EVs or *Dd*EVs may be responsible for some of the subsequent differences observed in the cytokine profiles. In fact, it has been reported that TGF‐β production (whose levels do not depend on NF‐κβ) is a result of a different signalling cascade involving Smad proteins (Derynck et al., 1998). NF‐κβ expression is not affected after a 24 h *Fh*EV treatment, but NOS2 mRNA levels were increased. NOS2 expression is dependent on NF‐κβ activation, but this regulation is reciprocal because the transcription of NF‐κβ can be also inhibited by NOS2 (Kelleher et al., 2007). Accordingly, after 24 h of treatment with *Dd*EVs, NOS2 levels were decreased and NF‐κβ levels were increased. It is possible that an early induction of NF‐κβ by *Fh*EVs could be responsible for the increase in NOS2 expression observed in macrophages.

Interestingly, both *Dd*EVs and *Fh*EVs reduced the expression of the M1 macrophage marker CD80, but only treatment with *Fh*EVs lead to an increase in the expression of CD206, the M2 macrophage marker. The increase in NOS2 and CD206 expression has also been observed in previous reports studying macrophages from infected animals, suggesting that *Fh*EVs could act as key mediators involved in the ability of *F. hepatica* to modulate the host immune response towards a Th2 type (Ruiz‐Campillo et al., 2018). *Fh*EVs seem to modulate THP‐1 macrophages towards a phenotype characterized by high levels of TGF‐β and CD206 expression (M2). *F. hepatica* produces an extensive disorganization of the hepatic architecture due to the scars from repaired migratory tracks (Rahko, 1972). Since M2 macrophages are crucial in tissue repair and fibrosis, the observed phenotype in macrophages treated with *Fh*EVs, characterized by an increased expression of TGF‐β, could be responsible for promoting liver fibrosis, a typical process observed in *F. hepatica* infections. However, after a 24 h treatment, we could not detect expression of other well‐known M2 markers in helminth infections like IL‐10, ARG1 or RETN (Gazzinelli‐Guimaraes & Nutman, 2018; Yunna et al., 2020). Parasitic helminths regulate the host immune response leading to chronic or long‐term infections, therefore, it is probable that longer co‐incubation times with EVs would modulate macrophages towards a more markedly M2 phenotype.

As far as we know, this study is the first report describing some functional properties of *Dd*EVs as immunoregulators. Very recent histological studies have suggested the central role of macrophages in the modulation of the inflammatory response and remodelling of the liver during infections by *D. dendriticum* (Piegari et al., 2021). In this context, our results highlight *Dd*EVs as important mediators of the immune response produced by *D. dendriticum* infections.

To further investigate the role of macrophages in the anti‐inflammatory properties described previously for *Fh*EVs (Roig et al., 2018) and compare with the modulation exerted by *Dd*EVs, we have analyzed the effect of both, *Fh*EVs and *Dd*EVs on LPS‐activated macrophages. In differentiated THP1‐XBlue™ CD14 cells to macrophages, LPS has been shown to induce a fast and powerful production of pro‐inflammatory cytokines such as IL‐1β, TNF‐α or IL‐6, through the TLR4‐NF‐κβ signaling pathway, producing its classical activation (Gordon, 2003). The administration of *Fh*EVs or *Dd*EVs produced a different response, as only macrophages treated with *Fh*EVs showed a reduction of NF‐κβ levels, but no differences in the cytokine profile were observed after the 24 h treatment. Nevertheless, after 48 and 72 h treatments with *Fh*EVs, expression changes were detected, indicating a sequential mechanism of action that may explain how *Fh*EVs inhibit inflammation. We have shown that LPS produce a marked increase in the expression of TNF‐α, TGF‐β, IL‐1β and CXCL8. The treatment with *Fh*EVs decreased the IL‐1β, IL‐6 and CXCL8 levels after 48 h of treatment and increased TGF‐β and IL‐13 expression after 72 h, not affecting TNF‐α expression. IL‐13 has been shown to inhibit the production of many inflammatory cytokines. This cytokine is mainly produced by different T‐cell subsets and DCs, although its expression in macrophages has also been reported (Hancock et al., 1998). TGF‐β is a pleiotropic cytokine which regulates many cellular processes and it is often considered as an anti‐inflammatory cytokine (Sanjabi et al., 2009). The observed reduction of IL‐6 in cells treated with *Fh*EVs suggests an anti‐inflammatory action of this cytokine. IL‐10 did not show any significant variation upon LPS stimulation with or without *Fh*EV treatment. These results are consistent with previous in vivo approaches using C57BL/6 and RAG1^−/−^ mice showing that IL‐10 was not involved in the protective effect on DSS‐induced colitis (Roig et al., 2018).

To identify protein targets that might be involved in the early modulatory effect on macrophages, we have studied the proteomic content of differentiated THP1‐XBlue™ CD14 cells after 24 h of *Fh*EV treatment in non‐treated and LPS‐activated macrophages. The levels of BAK1 and RAC1 proteins were increased in macrophages in the absence of LPS after the treatment with *Fh*EVs. Previous studies have reported that *F. hepatica* ESP can induce apoptosis in innate immune cells such as macrophages (Guasconi et al., 2012). The BAK1 protein plays a role in mitochondrial‐mediated apoptotic processes, and it was the protein with the highest increase after the treatment with *Fh*EVs. This, in addition to the modulation of macrophages towards an M2 phenotype, suggest a mechanism by which EVs may play a role ameliorating the immunopathology in the host avoiding the host immune response against this helminth. The RAC1 protein interacts with a wide range of effectors, playing a key role in cytoskeleton‐associated processes and inflammation (Payapilly & Malliri, 2018). In fact, RAC1 has been related to NF‐κβ expression, as it regulates the degradation of IκBα, an inhibitor of NF‐κβ (Kim & Yoon, 2016). In addition, RAC1 has been related to angiogenesis, which is a crucial step in new tissue generation, including wound healing (Fryer et al., 2005). As M2 macrophages are crucial for tissue‐repair, and probably in *F. hepatica*‐related liver fibrosis, RAC1 could be a key regulator induced by *Fh*EVs in this process.

Among the proteins whose levels are decreased upon *Fh*EV treatment, the PSMD6 protein, a component of the 26S proteasome, is one of the most interesting ones. The ubiquitin‐proteasome system is involved in a wide range of different processes, including inflammation and autoimmune diseases (Wang & Maldonado, 2006). Previous reports showed that down‐regulation of proteasome function is directly related to a decrease in the activation of the MAPK cascade, as well as TLR and TNF‐α expression (Qureshi et al., 2003). This downregulation of proteasome proteins may be an important mechanism by which *Fh*EVs modulate macrophages towards a regulatory phenotype *in* *vivo*.

To better understand the anti‐inflammatory effects of *Fh*EVs, the protein content of LPS‐activated macrophages upon *Fh*EV treatment was also analyzed. The number of differentially expressed proteins in LPS‐activated macrophages was lower than in LPS‐free conditions, probably due to the strong inflammatory phenotype produced by LPS. Under these conditions, *Fh*EVs induce the production of proteins directly involved in leukocyte migration and tissue repair, such as Galectin‐3 (Gao et al., 2014) and Reticulon‐4 (Yu et al., 2009). As *Fh*EVs promote the expression of TGF‐β, it is conceivable that these proteins contribute to modulate the infiltration of monocytes that ultimately lead to a decrease in the severity of the inflammation produced by the *Fh*EV treatment, as described previously (Roig et al., 2018). In fact, our results show that *Fh*EVs inhibit monocyte migration, whereas the EV‐depleted fractions, containing soluble proteins secreted by the parasite, (F17‐20 SEC) did not have the same effect, which suggests a selective packaging of metabolites into EVs depending on the parasites’ needs. Liver injuries can activate hepatic macrophages, which can either arise from circulating monocytes or from local macrophages, termed Kupffer cells. Circulating monocytes are probably not necessary to replenish the hepatic macrophage pool in homeostasis. However, hepatic damage may result in a massive infiltration of these circulating cells into the liver, exerting inflammatory actions (Tacke & Zimmermann, 2014). This modulation of monocyte migration might be an additional mechanism used by *F. hepatica* to alter the population of liver macrophages. This phenomenon seems to be species‐specific, as neither *Dd*EVs nor F17‐20 *Dd*SEC induced a significant variation on monocyte migration.

IFIT3 protein levels in macrophages are decreased by *Fh*EVs. This decrease is interesting since a macrophage polarization towards M1 phenotype, resulting in an increased expression of IFIT1, IFIT2, and IFIT3 proteins, has been described (Huang et al., 2018). Thus, its down‐regulation could limit LPS‐induced inflammation.

Regarding proteins not secreted through EVs, only few targets were affected upon *Fh*EV treatment, in both LPS‐activating and control conditions. The only protein with a decreased secretion in LPS‐activated macrophages was ANXA2, a calcium and phospholipid binding protein with a pleiotropic role in inflammation. In the early stages of inflammation, ANXA2 is important for the recruitment of some types of leukocytes, such as neutrophils, presenting a potential role in macrophage immunomodulation (Dallacasagrande & Hajjar, 2020).

In conclusion, our data show that EVs from *F. hepatica* and *D. dendriticum* trigger different responses in monocytes and macrophages, confirming their distinct role to either promote or inhibit the inflammatory response depending on the parasite source of EVs. *Fh*EVs lead macrophages to a mixed M1/M2 response, exerting anti‐inflammatory properties in LPS‐activated macrophages, while *Dd*EVs seem to elicit pro‐inflammatory properties. Furthermore, in this study, we have shown the role of macrophages in the immune modulatory response to *Fh*EVs, identifying several proteins that might be involved in the *Fh*EV‐macrophage interaction. All these features might correspond to the differences in the life cycle of both parasites and to the different histopathology observed. Future studies will address whether these differences are also reproducible in liver cells. Taken together, our results add new information and open new lines of investigation to fully understand the parasitosis caused by trematodes.

## AUTHOR CONTRIBUTIONS

**Christian M. Sánchez‐López**: Conceptualization; Formal analysis; Investigation; Methodology; Validation; Visualization; Writing—original draft; Writing—review editing. **Aránzazu González‐Arce**: Data curation; Methodology. **Carla Soler**: Methodology; Validation; Writing –review editing. **Victor Ramírez‐Toledo**: Methodology; Resources. **María Trelis**: Supervision; Validation; Writing Original Draft; Writing –review editing. **Dolores Bernal**: Supervision; Validation; Writing—original draft; Writing—review editing. **Antonio Marcilla**: Conceptualization; Funding acquisition; Project administration; Supervision; Writing—original draft; Writing—review & editing.

## CONFLICT OF INTEREST STATEMENT

The authors declare no conflict of interest.

## Supporting information

Supplementary Table 1. List of proteins identified in *Fasciola hepatica* EVs by LC‐MS/MS.

Supplementary Table 2. List of proteins identified in *Dicrocoelium dendriticum* EVs by LC‐MS/MS.

Proteins in THP‐1 cells treated with LPS and *Fh*EVs not identified in THP‐1 cells treated with LPS
